# Aspiration Pneumonia After Lumbar Spinal Intervention

**DOI:** 10.7759/cureus.103381

**Published:** 2026-02-10

**Authors:** Hyeryung Kang, Yoojeong Kwak

**Affiliations:** 1 Department of Anesthesiology and Pain Medicine, Veterans Health Service Medical Center, Seoul, KOR

**Keywords:** aspiration pneumonia, iatrogenic disease, immobility, infectious spondylitis, intervertebral disc disease, low back pain

## Abstract

Low back pain is a prevalent complaint among patients, leading to the frequent use of various interventional procedures in pain clinics. While these interventions are generally regarded as safe, complications, particularly in elderly patients, can include serious conditions such as infectious spondylitis or spinal hematoma. We report a case of an elderly patient who developed aspiration pneumonia shortly after undergoing a lumbar spinal intervention. The initial clinical suspicion was infectious spondylitis due to the temporal association with the procedure. However, further evaluation revealed that the procedure itself was not the direct cause. Instead, the exacerbation of low back pain following the intervention likely resulted in prolonged bed rest and ineffective coughing, which contributed to the worsening of pre-existing pneumonia. This case highlights the importance of considering aspiration pneumonia as a potential complication following lumbar procedures in elderly patients, especially when symptoms, such as fever and respiratory distress, occur post-intervention. Distinguishing aspiration pneumonia from other serious complications, such as infectious spondylitis, is crucial for appropriate management. Pain clinicians should maintain a high index of suspicion for such complications and exercise caution when treating elderly individuals. Aspiration pneumonia can develop or worsen after lumbar interventions in elderly patients, particularly when post-procedural pain leads to decreased mobility and impairs airway clearance. Clinicians should be vigilant for respiratory complications and carefully differentiate them from procedure-related infections to ensure optimal patient outcomes.

## Introduction

Low back pain is a prevalent condition affecting 619 million people globally in 2020 [1]. Thirty-three percent of chronic low back pain patients are elderly (>65 years), necessitating caution during interventional procedures. Although lumbar procedures are generally safe, complications, including hematoma (0.5-1%), infectious spondylitis (<1%), or rare paraplegia, can occur. Careful patient selection and monitoring minimize these risks [2].

## Case presentation

An 81-year-old male (height: 154 cm, weight: 61 kg) presented to the pain clinic with chronic low back pain and claudication of the lower extremities. His medical history included hypertension, stage I lung adenocarcinoma treated with video-assisted thoracoscopic surgery (VATS) right lower lobectomy and adjuvant chemotherapy five years prior, and a subtotal gastrectomy for gastric cancer 10 years prior. The lung surgery history is relevant to his baseline aspiration risk due to a potentially impaired cough reflex. He was also receiving medications for benign prostatic hyperplasia and depression.

Approximately three months prior to the lumbar procedure, the patient presented to the outpatient pulmonology clinic with a cough and sputum production. Chest X-ray revealed diffuse reticular opacity in the right lower lung field (RLLF), and he was diagnosed with pneumonia. He received a one-week course of oral cefditoren, after which his symptoms and radiographic findings improved.

**Figure 1:**
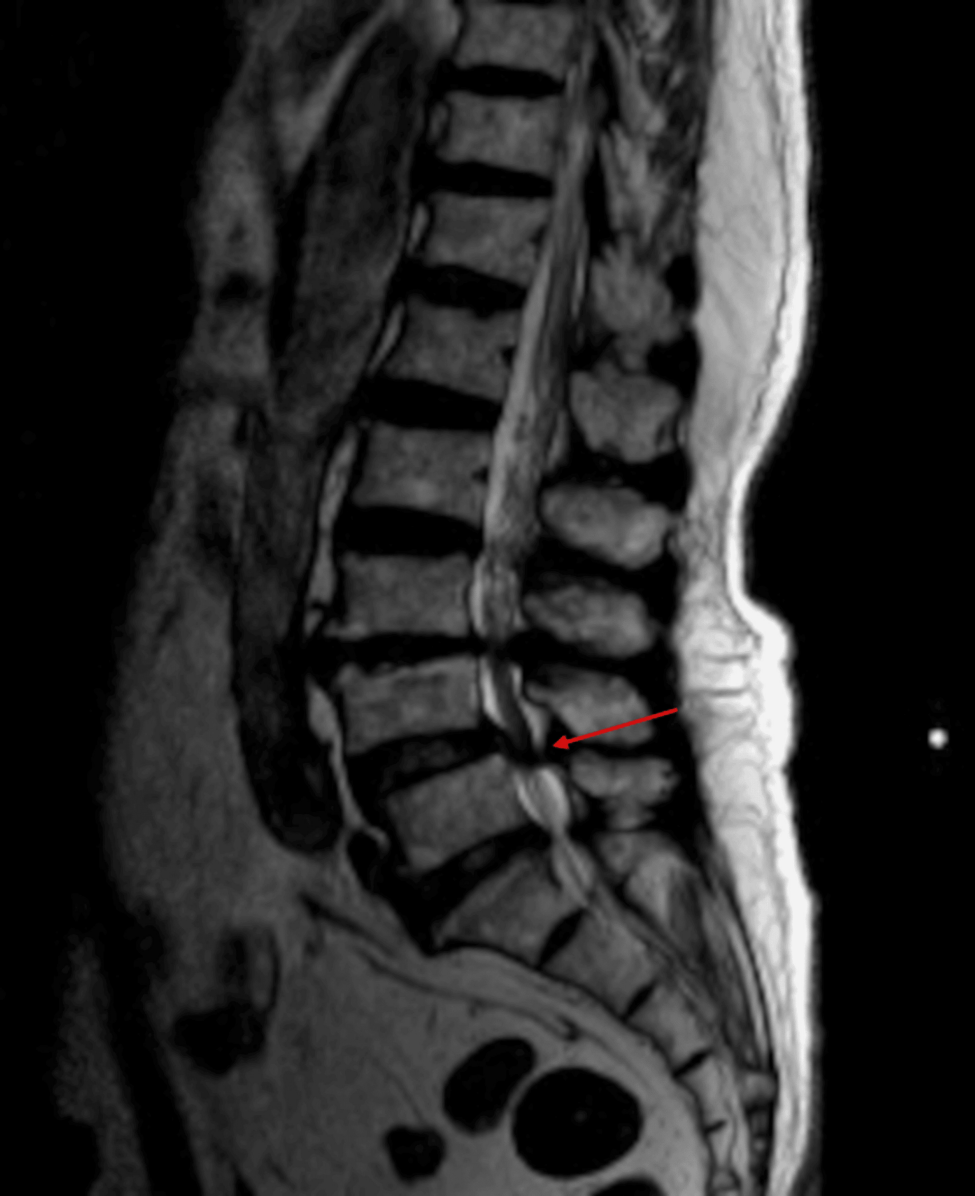
Lumbar spine MRI (T2) taken before the procedure (sagittal view). The red arrow indicates the area of severe central stenosis at the L4/5 level. Sagittal view of lumbar spine magnetic resonance imaging (L-MRI) showing severe central stenosis at the L4/5 level, as indicated by the red arrow.

**Figure 2:**
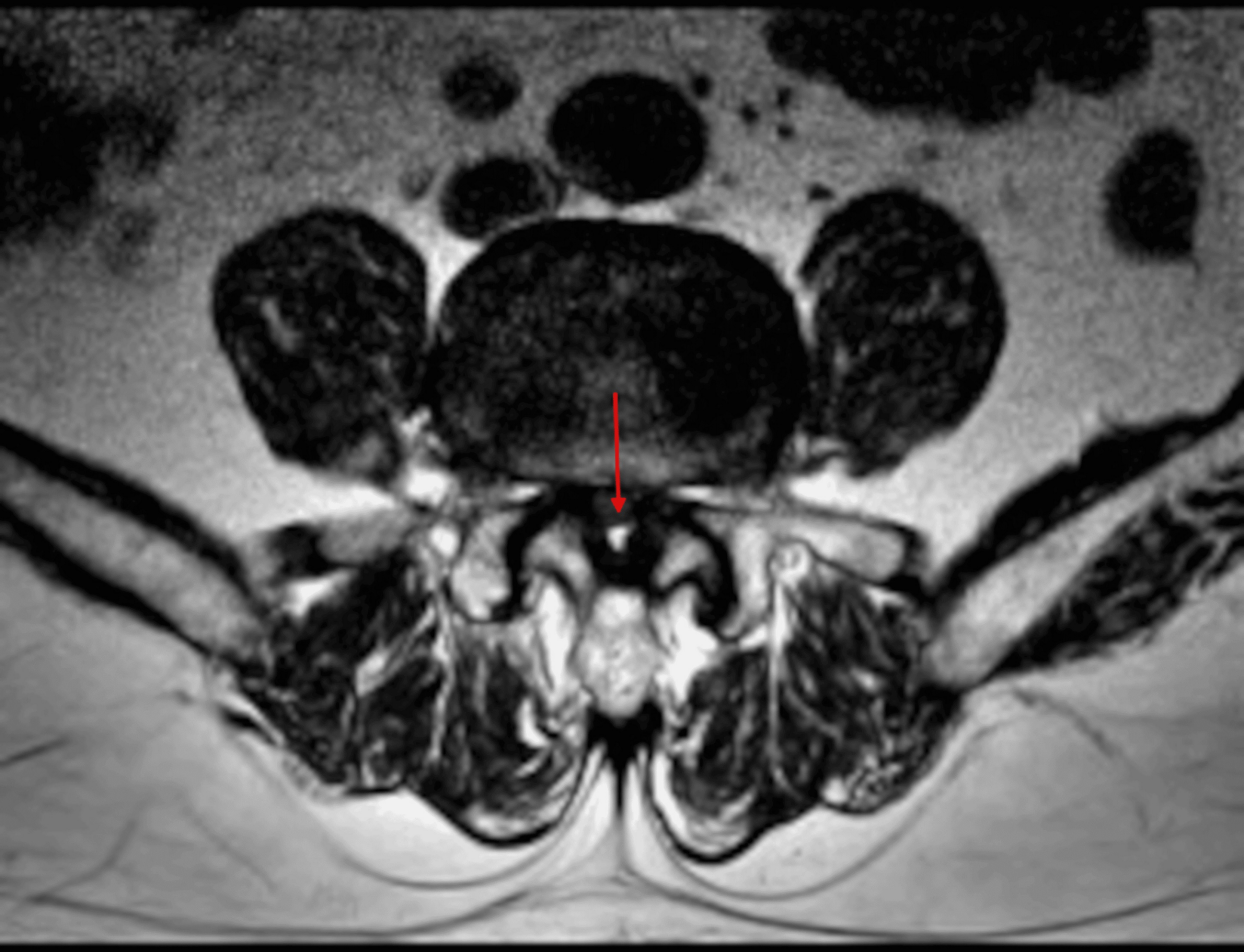
Lumbar spine MRI (T2), axial view before the procedure. The red arrow indicates severe central canal stenosis at L4/5. Axial T2-weighted MRI of the lumbar spine demonstrating markedly narrowed central spinal canal at the L4/5 level, as indicated by the red arrow.

Lumbar spine magnetic resonance imaging (L-MRI) demonstrated severe central stenosis at the L4/5 level (Figures 1, 2). Previous treatments, including medications (gabapentin, limaprost, and tramadol), multiple lumbar epidural steroid injections, and percutaneous epidural neuroplasty, had failed to provide adequate relief. Therefore, intradiscal electrothermal therapy (IDET) was performed to address his low back pain.

**Figure 3:**
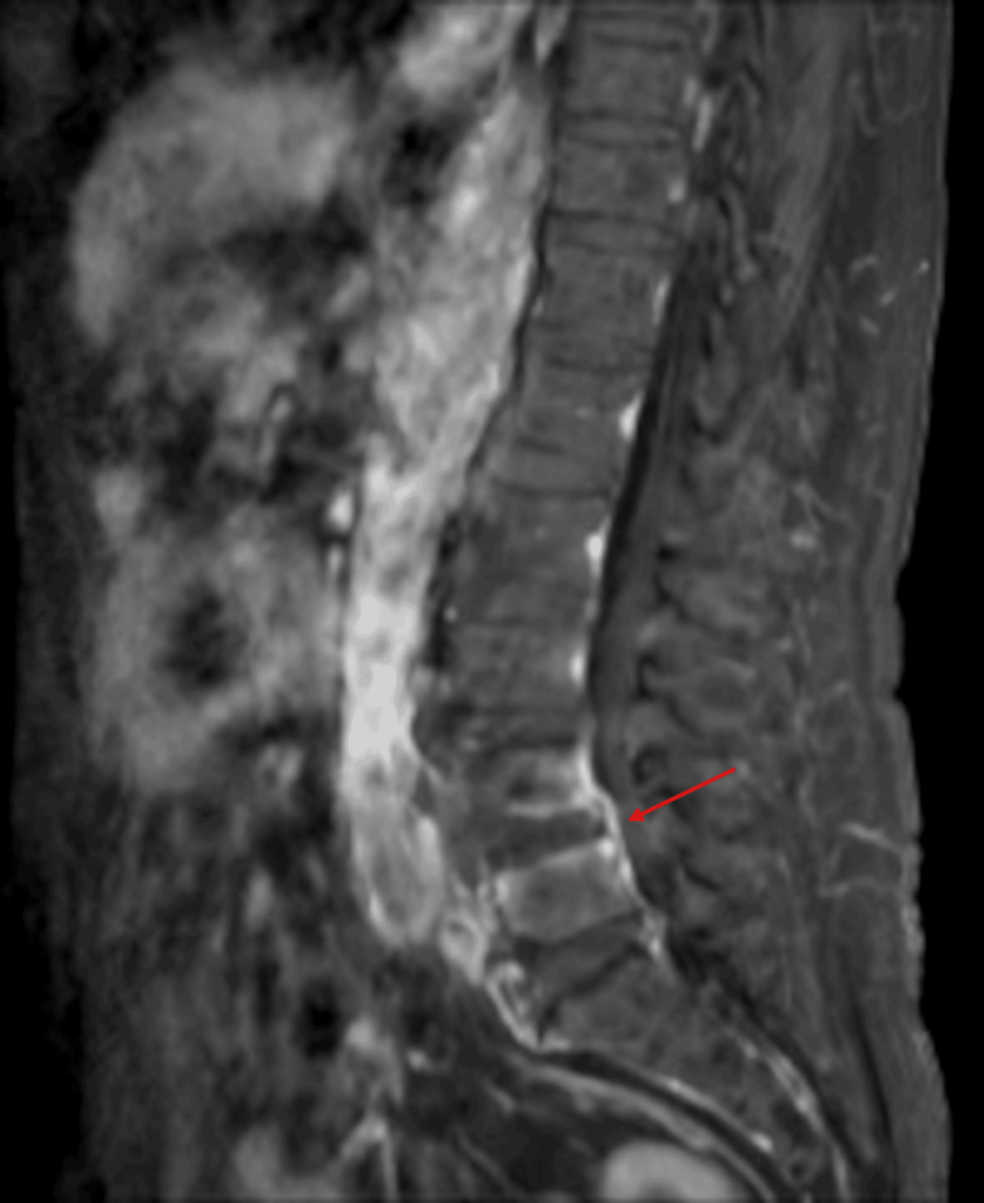
Contrast-enhanced lumbar MRI two weeks post-procedure. The red arrow indicates enhancing soft tissue in the anterior epidural space at L4/5. The red arrow highlights mildly thickened, enhancing soft tissue in the ventral epidural space located just anterior to the dura at the L4/5 level. This faintly brightened area may represent inflammatory, infectious, or reactive changes.

The day after IDET, the patient returned to the clinic with severe back pain, rendering him unable to stand and confining him to bed. After one week of immobilization, laboratory tests showed elevated inflammatory markers (erythrocyte sedimentation rate {ESR}: 42 mm/h; C-reactive protein {CRP}: 23.81 mg/L). Five days later, follow-up blood tests revealed further elevation (ESR: 94 mm/h; CRP: 140.71 mg/L), and contrast-enhanced lumbar MRI demonstrated subtle contrast enhancement at the L4/5 endplate and mild thickening of the enhancing soft tissue in the anterior epidural space at L4/5 (Figure 3). The radiologist suggested a differential diagnosis of early infectious spondylitis versus reactive changes. During this period, the patient developed severe cough and hemoptysis in addition to worsening back pain.

**Figure 4:**
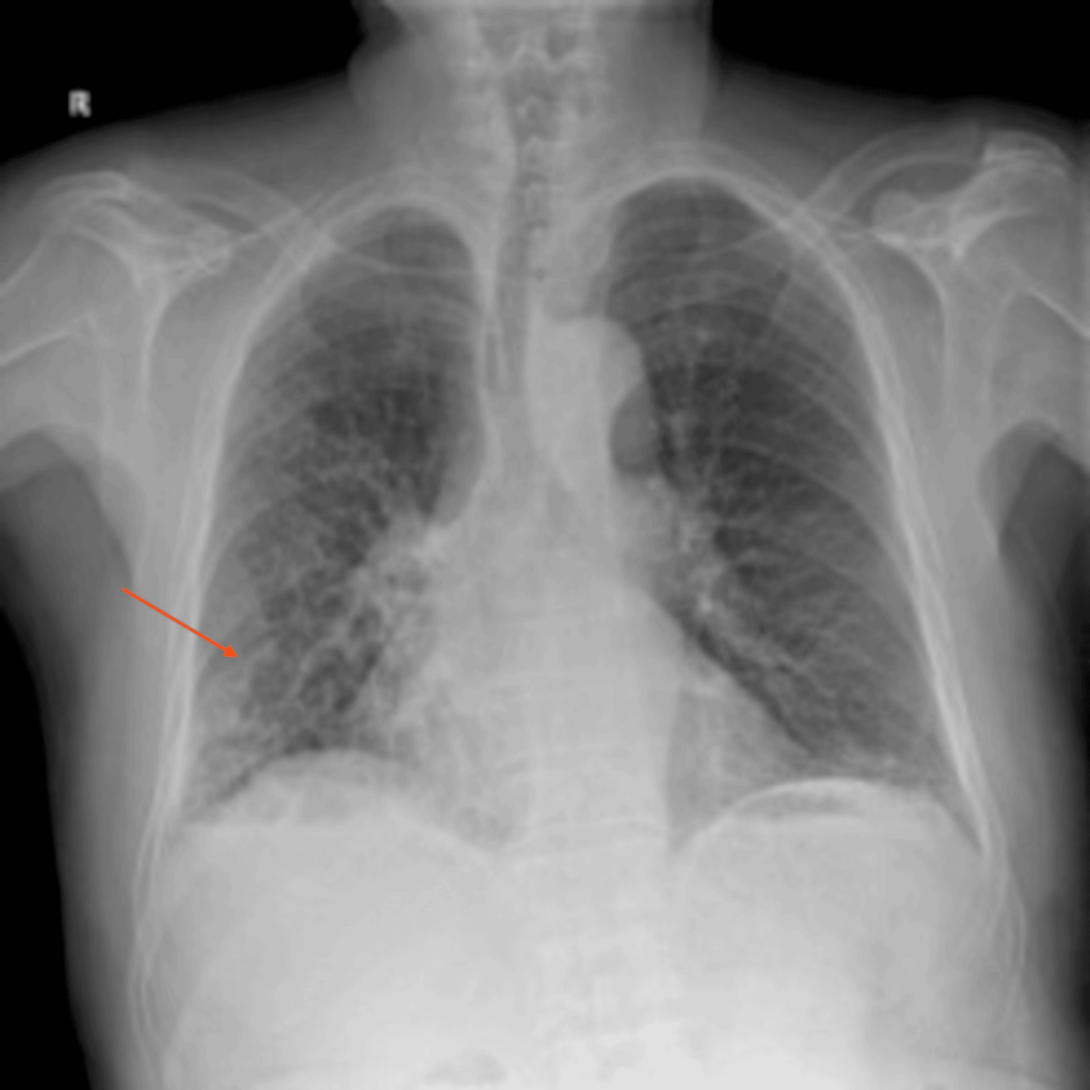
Post-admission chest X-ray showing increased opacity in the right lower lung field, indicated by the red arrow. Chest X-ray revealing diffuse aspiration bronchiolitis: red arrow (RLLF consolidation); additional multilobar findings (tracheal wall sputum, left lower lobe infiltrate, right upper/middle infiltrates) - characteristic of chronic silent aspiration in frail elderly. RLLF: right lower lung field

**Figure 5:**
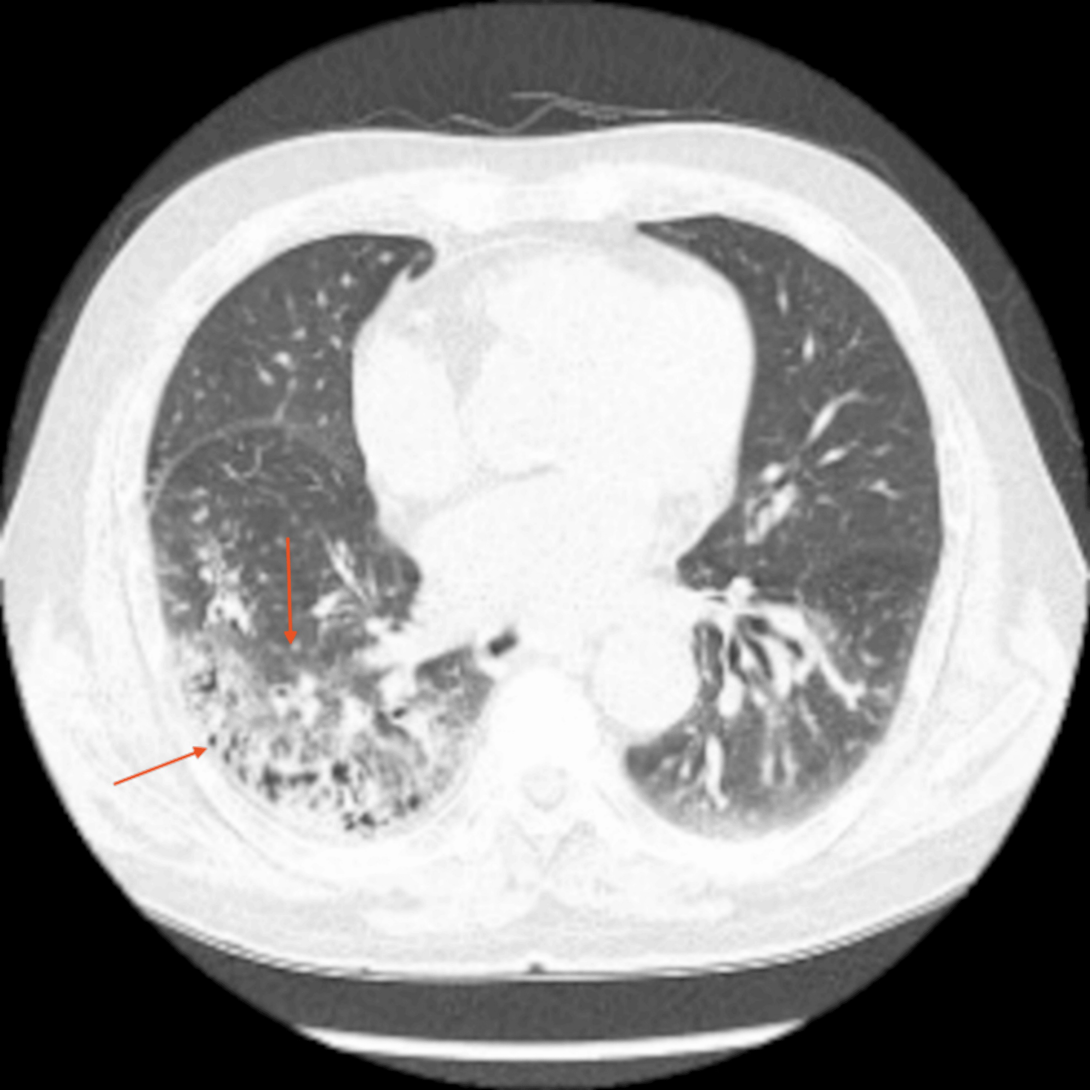
Chest CT suggesting aspiration pneumonia. The red arrow highlights consolidation and ground-glass opacity in the right lower lobe. Axial view of chest CT demonstrating an area of consolidation and ground-glass opacity in the right lower lobe, consistent with aspiration pneumonia. The red arrow marks the region of interest.

The patient was admitted for further evaluation. Additional diagnostic workup, including chest X-ray, blood, sputum, and stool cultures, was performed. Chest X-ray showed opacity in the right lower lung field (RLLF), and chest CT confirmed aspiration pneumonia (Figures 4, 5). Blood, sputum, and stool cultures were negative. After respiratory consultation, ceftriaxone 2 g IV q24h plus metronidazole 500 mg IV q8h was initiated for 10 days (followed by oral amoxicillin-clavulanate), with clinical/radiologic improvement. After consultation with the internal medicine respiratory team, antibiotic therapy for aspiration pneumonia was initiated.

**Figure 6:**
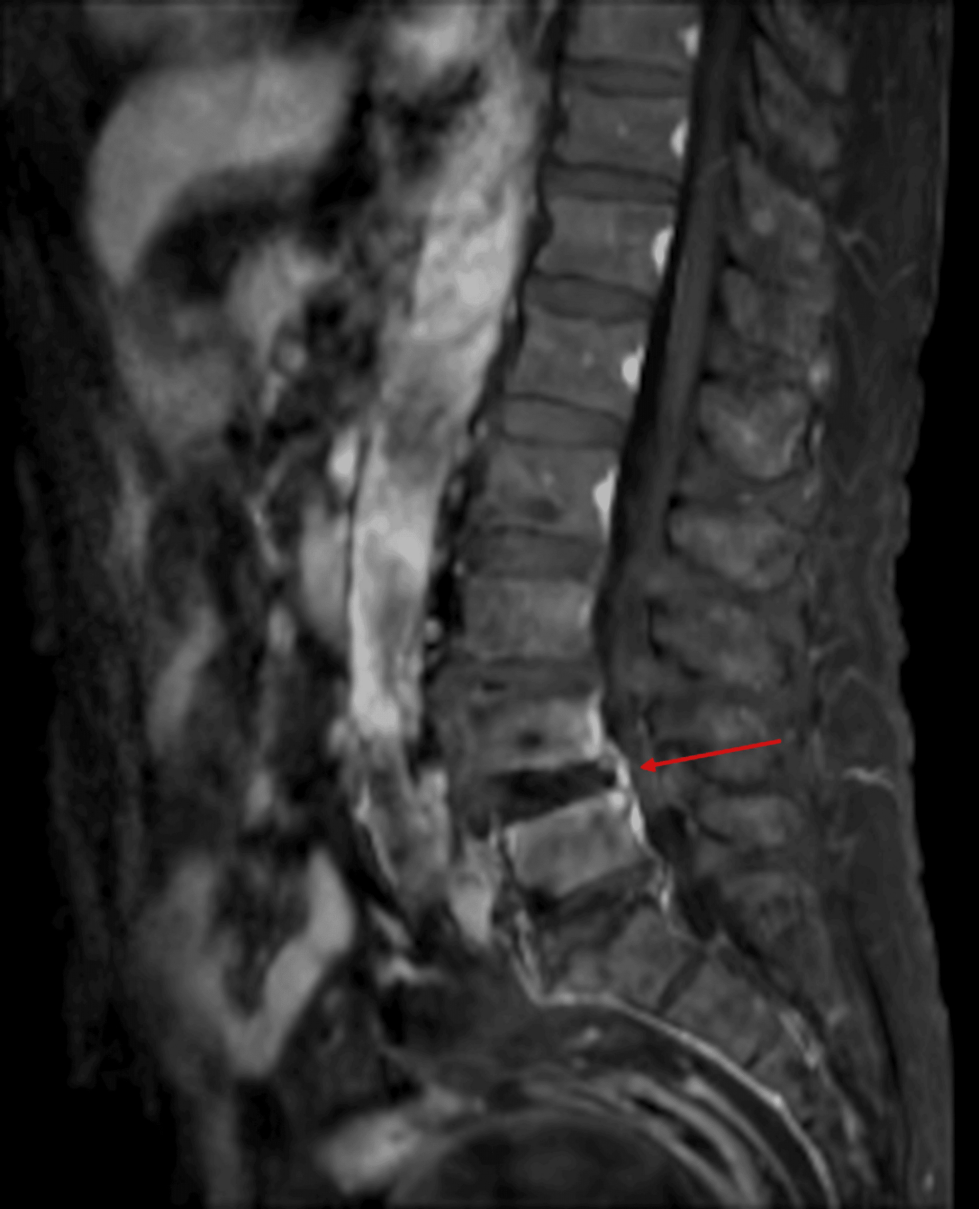
Follow-up contrast-enhanced lumbar MRI 19 days after the procedure. The red arrow indicates mildly thickened enhancing soft tissue in the anterior epidural space at L4/5. Contrast-enhanced lumbar MRI taken 19 days after the procedure. The red arrow highlights mildly thickened, enhancing soft tissue in the anterior epidural space at the L4/5 level, showing no significant interval change from the previous scan.

A follow-up contrast-enhanced L-MRI, performed 19 days after the lumbar procedure, demonstrated a slight reduction in contrast enhancement at the L4/5 endplate and no significant change in the mild thickening of the enhancing soft tissue in the anterior epidural space at L4/5. The radiologist’s report favored improving reactive change over early infectious spondylitis (Figure 6). Clinically, the patient’s low back pain improved significantly, although cough and sputum production persisted. Inflammatory markers also improved (ESR: 63 mm/h; CRP: 32 mg/L), and the patient was discharged following clinical and laboratory improvement. Lung cancer relapse was excluded based on cytology and clinical stability.

## Discussion

Low back pain (LBP) is a prevalent condition, with a reported lifetime prevalence of 84% in the general adult population [1]. In elderly individuals, LBP often leads to restrictions in activities of daily living, prompting them to seek medical treatment [2]. Management options for LBP include conservative approaches, such as medications and physical therapy, as well as interventional procedures like lumbar epidural steroid injections and other minimally invasive techniques. While these interventional procedures are effective for many patients, they are associated with a range of complications. Most are minor, including vascular penetration, post-procedural headaches, transient exacerbation of back or leg pain, facial flushing, intraoperative hypertension, transient nerve root irritation, and vasovagal reactions, with an incidence ranging from 2.4% to 9.6%. However, major complications, though exceedingly rare, can have devastating consequences, such as epidural abscess, epidural hematoma, spinal cord infarction, infectious spondylitis, and inadvertent dural or subdural puncture [3]. The majority of these complications are attributed to direct needle injury or corticosteroid injection.

This case illustrates post-procedural immobilization following lumbar intervention, associated with the development of aspiration pneumonia, creating a diagnostic dilemma given that inflammatory markers and MRI findings initially mimicked infectious spondylitis. While pneumonia is not a commonly reported complication of such procedures and may have a limited direct causal relationship, we believe that this was not merely an incidental occurrence. Although lumbar procedures are generally safe, complications including neurological injury (0.5-1%), hematoma (<0.1%), or infection (<0.1%) can occur [2]. Careful patient selection and monitoring minimize these risks. This highlights the need for respiratory evaluation in elderly patients with fever following lumbar interventions. As demonstrated in this case, patients undergoing invasive lumbar procedures often experience increased pain at the procedural site for several days. However, it is important to consider that exacerbated post-procedural pain may play a role in the worsening of pre-existing pneumonia. Severe pain can significantly limit ambulation, particularly in elderly patients, leading to prolonged bed rest and difficulty with mobilization. Additionally, pain-related discomfort may impair effective coughing and deep breathing, hindering adequate pulmonary hygiene and expectoration. These factors, combined with reduced mobility and impaired lung care rehabilitation, can increase the risk of aspiration and subsequent pneumonia [4].

Another important consideration is to conduct a thorough history to assess whether the patient had any symptoms prior to the procedure. In frail elderly patients, chronic silent microaspiration during sleep represents the primary mechanism of aspiration pneumonia [5]. Post-lumbar-intervention immobilization exacerbates this baseline risk by impairing the cough reflex, causing muscle weakness, and reducing oral hygiene, necessitating prevention strategies beyond nothing by mouth (NPO) status (e.g., early mobilization, swallow assessment, oral care protocols). The patient had been treated for community-acquired pneumonia three months before the lumbar procedure, with subsequent symptom resolution and normalization of chest X-ray findings. However, it is possible that the pneumonia had not been completely eradicated at the time of the procedure. Incomplete adherence to antibiotic regimens or premature discontinuation of treatment may result in residual pulmonary inflammation, even in the absence of clinical symptoms, thereby predisposing the patient to pneumonia recurrence. Given this, clinicians performing invasive lumbar procedures should consider postponing the intervention if the patient presents with respiratory symptoms suggestive of a recent or chronic infection.

Respiratory symptoms may complicate the differentiation of post-procedural infectious complications, such as infectious spondylodiscitis. MRI with contrast serves as the key diagnostic tool by revealing abnormal signals in the affected intervertebral disc and helping to distinguish these conditions from others. However, respiratory symptoms may also lead to diagnostic uncertainty and inappropriate management. Moreover, the procedure itself could exacerbate pre-existing respiratory conditions. Prior to performing lumbar procedures in elderly patients, a thorough medical history, including a comprehensive review of systems, is essential. In some cases, laboratory tests such as complete blood count (CBC), serum chemistry, erythrocyte sedimentation rate (ESR), and C-reactive protein (CRP) may be warranted. However, opinions may differ regarding the necessity of routine blood tests for all patients before the procedure. While routine testing may enhance patient safety, it could also contribute to increased healthcare costs, patient anxiety, and false-positive results that might lead to unnecessary further investigations [6,7]. Further research is warranted to establish optimal pre-procedural screening strategies.

In the present case, intradiscal electrothermal therapy (IDET) failed to achieve optimal outcomes, highlighting the potential limitations of the procedure and the associated complications in elderly patients. IDET is a minimally invasive technique that uses a navigable intradiscal catheter to apply heat directly to the annulus of the intervertebral disc, aiming to reduce discogenic pain. However, when disc degeneration is severe, IDET becomes contraindicated, and its indications are generally limited [8]. Given that most elderly patients exhibit degenerative disc changes, it can be concluded that very few elderly patients meet the criteria outlined in the guidelines. The patient in this case presented with chronic axial back pain unresponsive to various conservative treatments, and the MRI showed only mild disc degeneration relative to his age. After thoroughly explaining the procedure and its potential side effects, the IDET procedure was performed. However, as seen in the post-procedure contrast-enhanced MRI, reactive changes suggestive of inflammatory and edematous alterations were observed in the epidural space around the L4/5 disc. These changes may have exacerbated pre-existing spinal stenosis, potentially aggravating his back pain.

The most important consideration for minimizing complications after procedures in elderly patients is encouraging early ambulation. The complications associated with prolonged immobility have been widely reported in elderly patients [9-11]. In this case, potential complications include worsening respiratory infections, cardiovascular events (e.g., arterial and venous thrombosis), urinary tract infections, and musculoskeletal issues such as weakness of the lower limbs and spinal muscles [12-16]. Major complications arising from immobility are significantly correlated with a decrease in health-related quality of life. In cases where pulmonary complications arise post-procedure, as in this case, a multifaceted approach to chest physiotherapy is recommended to prevent further issues. This regimen typically includes techniques such as coughing exercises, postural drainage, and incentive spirometry. These interventions aim to maintain airway patency, promote effective gas exchange, and reduce the risk of respiratory infections in immobilized patients. The implementation of such preventive strategies is crucial for managing patients at risk of respiratory complications due to immobility. Pain clinicians should exercise caution when performing lumbar procedures, particularly in elderly patients, because procedure-related pain can limit mobility and increase the risk of complications, including pneumonia.

## Conclusions

This study demonstrates the pathway as follows: lumbar interventions → immobilization → exacerbation of inevitable silent microaspiration → diagnostic mimicry of spondylitis. This case highlights that pneumonia can develop or worsen following lumbar interventional procedures in elderly patients, particularly when post-procedural pain leads to immobility and impaired pulmonary hygiene. Comprehensive pre-procedural assessment, careful patient selection, and early mobilization are essential to minimize the risk of such complications. Clinicians should maintain a high index of suspicion for respiratory complications when elderly patients develop fever and elevated inflammatory markers following lumbar procedures, and routinely obtain chest imaging to differentiate pneumonia from procedure-related infections, such as spondylitis.
